# Supplementary material for: An essential function for autocrine hedgehog signaling in epithelial proliferation and differentiation in the trachea
Source: Development. 2022 Feb 7;149(3):dev199804. doi: 10.1242/dev.199804 (PMC8918789; doi:10.1242/dev.199804)
Supplement: Supplementary information [file develop-149-199804-s1.pdf]

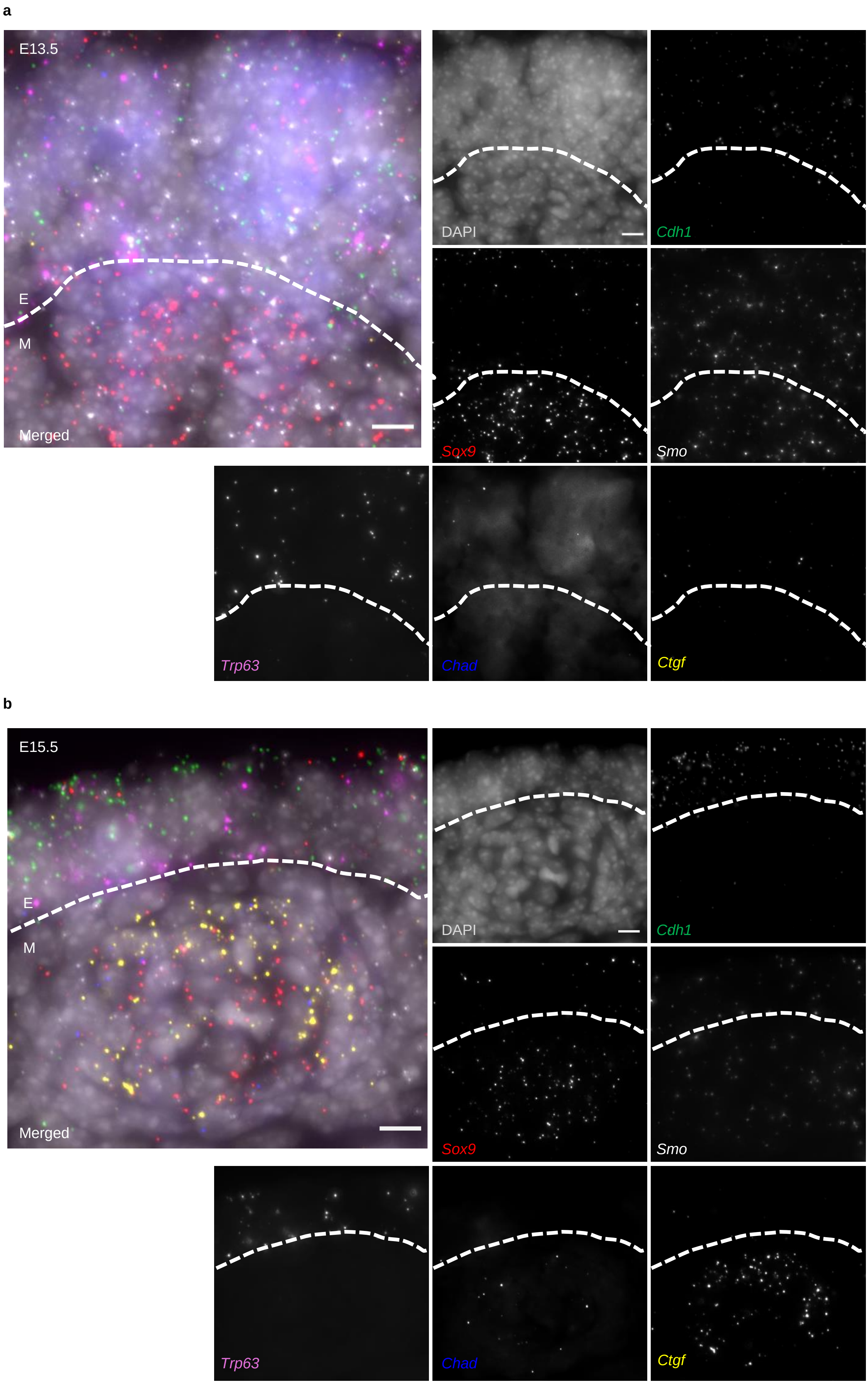
a
E13.5
E
M
Merged
DAPI
Cdh1
Sox9
Smo
Trp63
Chad
Ctgf
b
E15.5
E
M
Merged
DAPI
Cdh1
Sox9
Smo
Trp63
Chad
Ctgf

Fig. S1. *Smo* is expressed in tracheal chondroblasts at embryonic stages.
(**a**) mRNA *in situ* detection of *Smo* (white) in tracheal chondroblasts marked by the expression on *Sox9* (red), *Chad* (blue) *and Ctgf* (yellow) at E13.5. **(b)** mRNA *in situ* detection of *Smo* (white) in tracheal chondroblasts marked by the expression on *Sox9* (red), *Chad* (blue) *and Ctgf* (yellow) at E15.5. *Cdh1* (green) and *Trp63* (violet) mark the tracheal epithelium. Sections were counter stained with DAPI. Dashed lines indicate the border between epithelium (E) and mesenchyme (M). Scale bar: 5 μm.

Development • Supplementary information

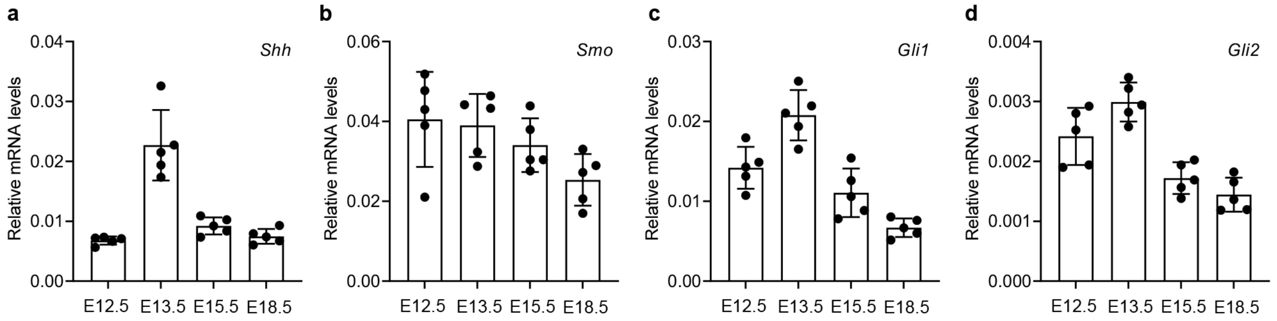

Fig. S2. *Shh, Smo, Gli1 and Gli2* are dynamically expressed in the developing trachea.

(a) RT-qPCR analysis of *Shh* mRNA levels in WT tracheae (n=5 per stage). **(b)** RT-qPCR analysis of *Smo* mRNA levels in WT tracheae (n=5 per stage). **(d)** RT-qPCR analysis of *Gli1* mRNA levels in WT tracheae (n=5 per stage). **(d)** RT-qPCR analysis of *Gli2* mRNA levels in WT tracheae (n=5 per stage).

Development • Supplementary information

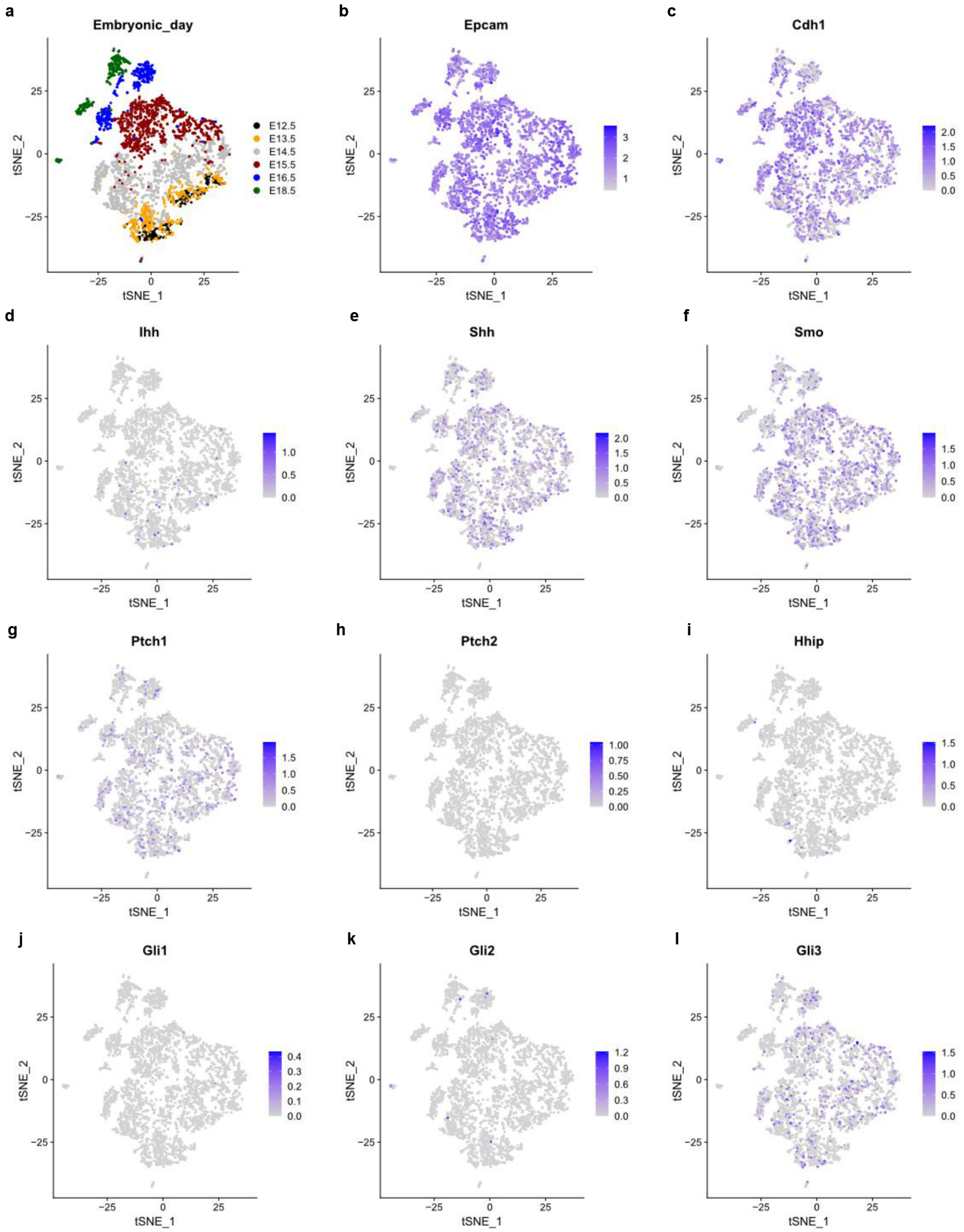

Development • Supplementary information

Fig. S3. Single cell analysis of Hh signaling components during development.

(**a**) t-SNE plots showing the developmental stage of embryonic tracheal cells from Kiyokawa et al. Dev. Cell 2021. (**b-l**) t-SNE plots showing the expression levels of Hh signaling components.

Development • Supplementary information

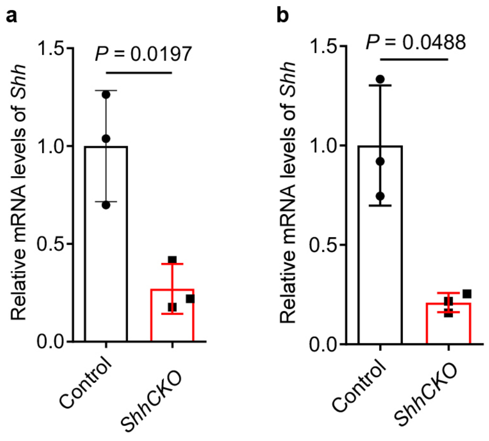

Fig. S4. *Shh* mRNA levels are dramatically reduced in *Nkx2.1^{Cre};Shh$^{flox/flox}$* tracheae. (a) RT-qPCR analysis of *Shh* mRNA levels in E11.5 control (n=3) and *ShhCKO* (n=3) tracheae. (b) RT-qPCR analysis of *Shh* mRNA levels in E18.5 control (n=3) and *ShhCKO* (n=3) tracheae.

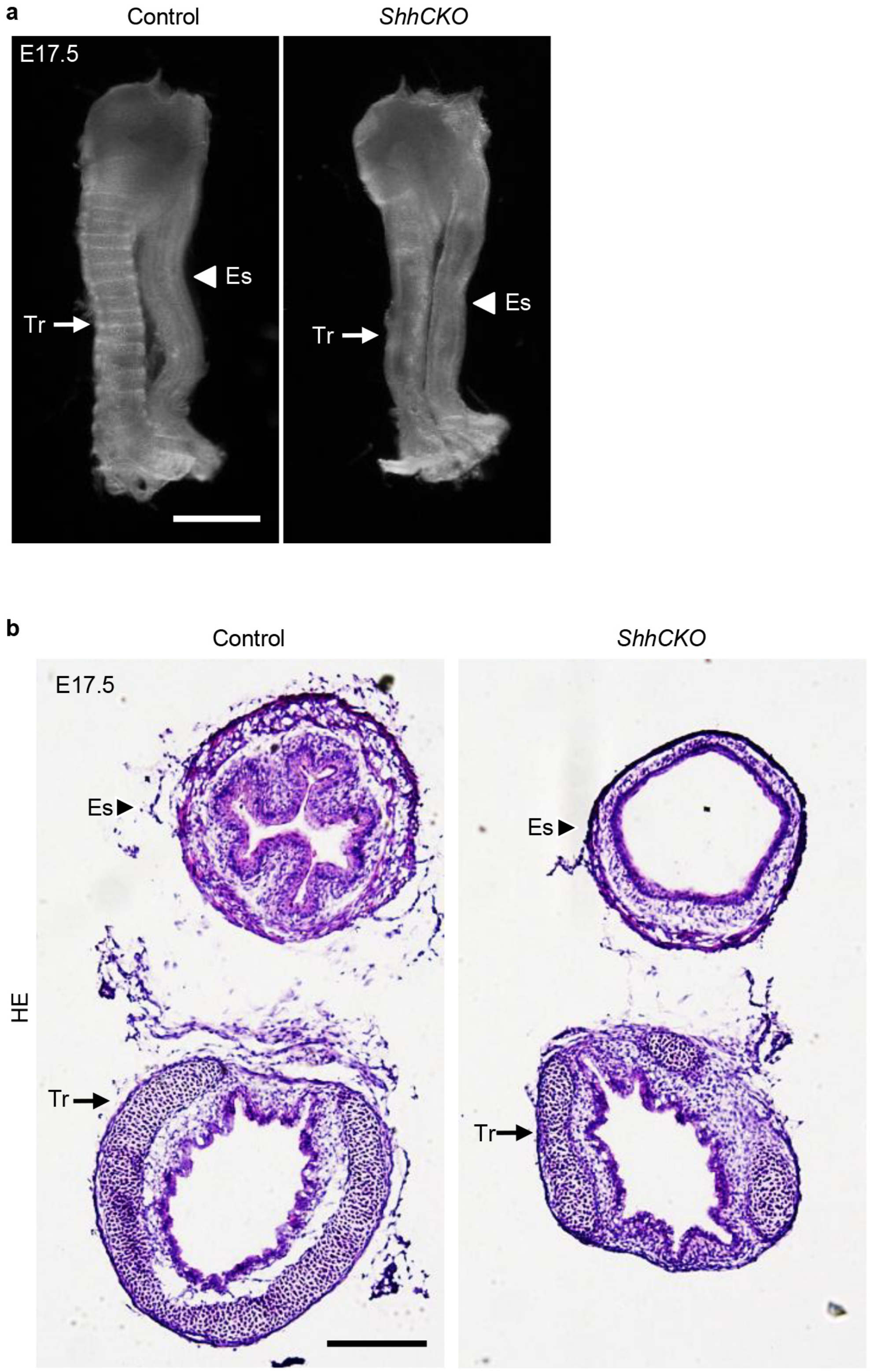

Fig. S5. ***Nkx2.1^{Cre};Shh$^{flox/flox}$*** **mice exhibit no trachea-esophageal fistula at E17.5.** (**a**) Representative images of E17.5 control (n=5) and *ShhCKO* (n=5) tracheae. Arrows point to the tracheae. Arrow heads point to the esophagus. (**b**) Representative images of tracheal and esophageal sections stained with hematoxylin and eosin from E17.5 control (n=5) and *ShhCKO* mice (n=5). Arrows point to the tracheae. Arrow heads point to the esophagus. Scale bars: 1000 µm (**a**), 200 µm (**b**). Tr, Trachea; Es, Esophagus.

Development • Supplementary information

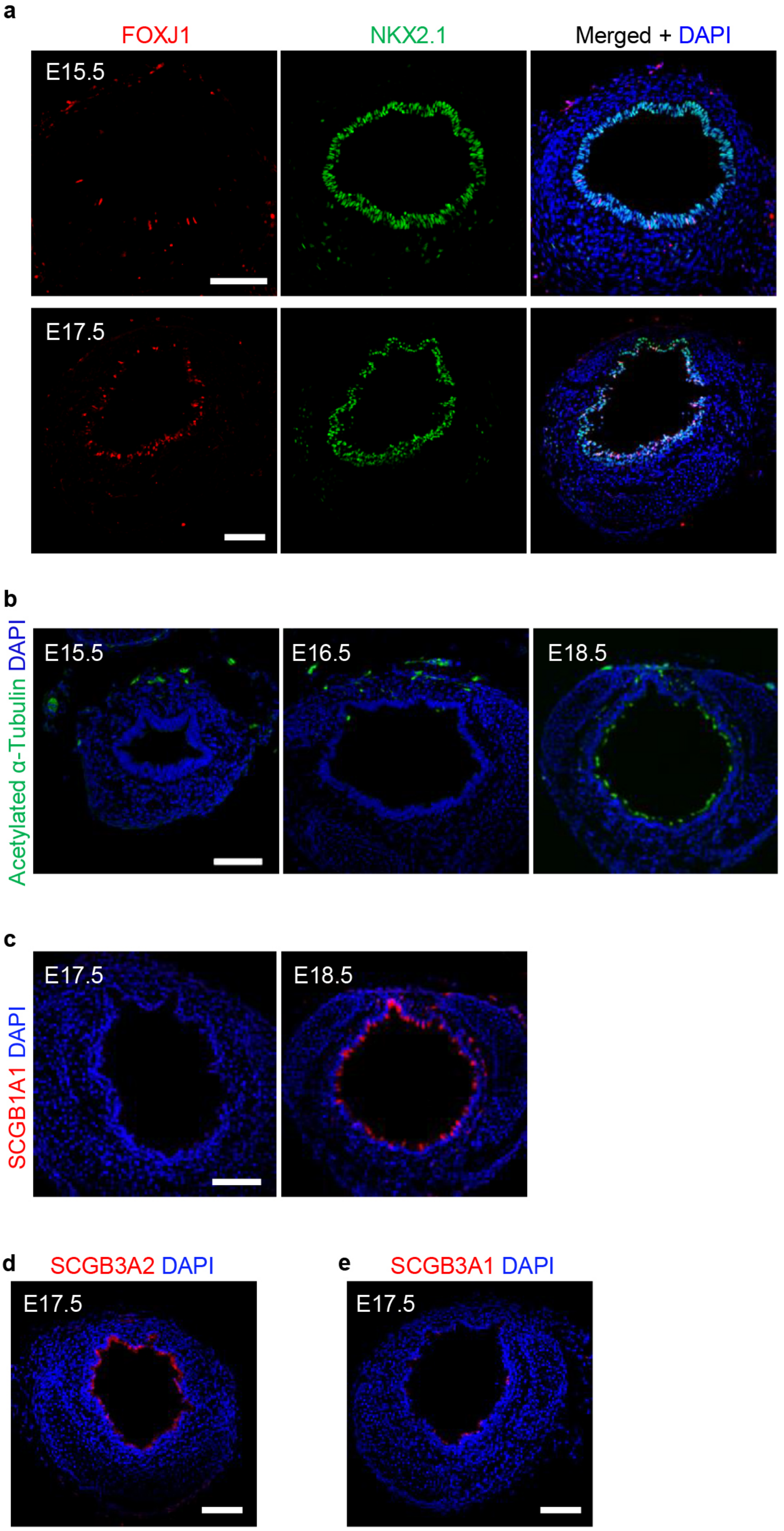

Development • Supplementary information

Fig. S6. Mature ciliated cells and club cells are widely distributed in the trachea at E18.5. (**a**) Immunostaining for FOXJ1 (red) and NKX2.1 (green), and DAPI staining (blue) of transverse sections of WT tracheae (n=7) at E15.5 and E17.5. **(b)** Immunostaining for acetylated alpha-tubulin (green) and DAPI staining (blue) of transverse sections of WT tracheae (n=7) at several embryonic stages. **(c)** Immunostaining for SCGB1A1 (red) and DAPI staining (blue) of transverse sections of WT tracheae (n=7) at several embryonic stages. **(d)** Immunostaining for SCGB3A2 (red) and DAPI staining (blue) of transverse sections of WT tracheae (n=7) at E17.5. **(e)** Immunostaining for SCGB3A1 (red) and DAPI staining (blue) of transverse sections of WT tracheae (n=7) at E17.5. Scale bars: 100 µm.

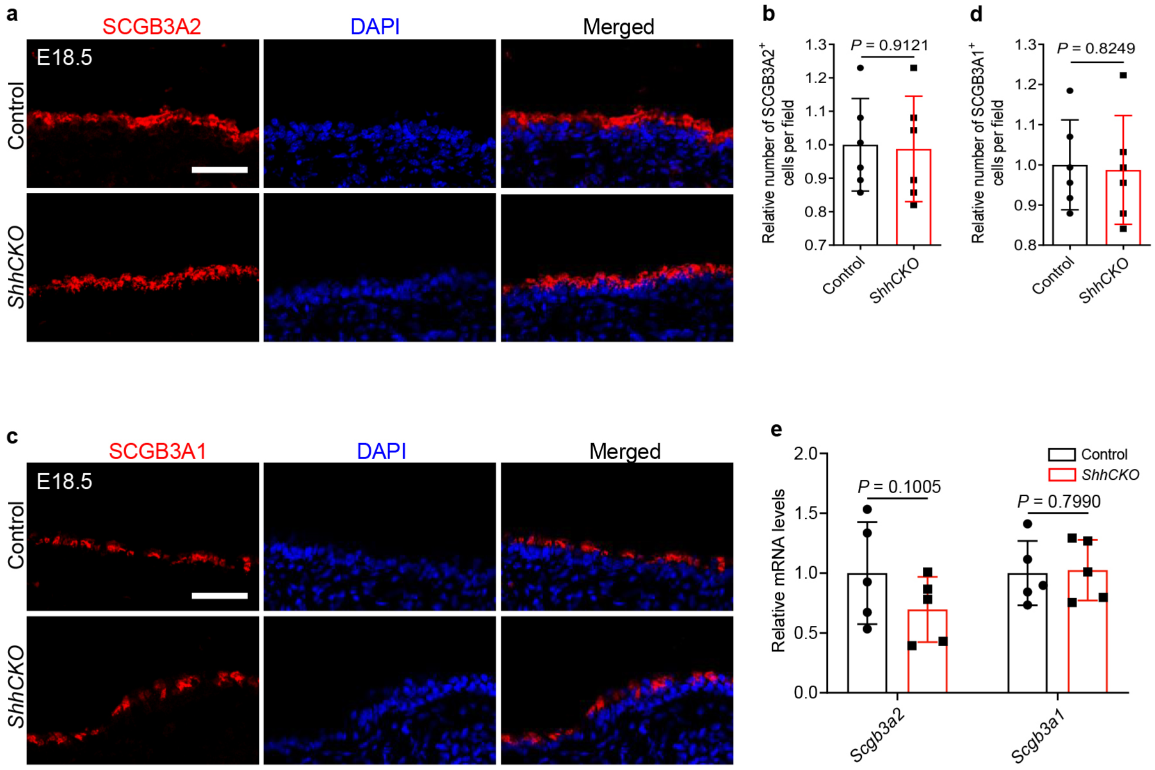

Fig. S7. SCGB3A2⁺and SCGB3A1⁺ cell differentiation is not affected in *Nkx2.1^{Cre}; Shh$^{flox/flox}$* mice.

(**a**) Immunostaining for SCGB3A2 (red) and DAPI staining (blue) of longitudinal sections of E18.5 control (n=6) and *ShhCKO* (n=6) tracheae. (**b**) Quantification of the relative number of SCGB3A2^{+} cells in the tracheal epithelium in control (n=5) and *ShhCKO* (n=5) mice. (**c**) Immunostaining for SCGB3A1 (red) and DAPI staining (blue) of longitudinal sections of E18.5 control (n=6) and *ShhCKO* (n=6) tracheae. (**d**) Quantification of the relative number of SCGB3A1^{+} cells in the tracheal epithelium in control (n=6) and *ShhCKO* (n=6) mice. (**e**) RT–qPCR analysis of *Scgb3a2* and *Scgb3a1* mRNA levels in E18.5 control (n=6) and *ShhCKO* (*n*=6) tracheae. Scale bars: 50 µm**.** Unpaired Student's *t*-test, mean ± s.d.

Development • Supplementary information

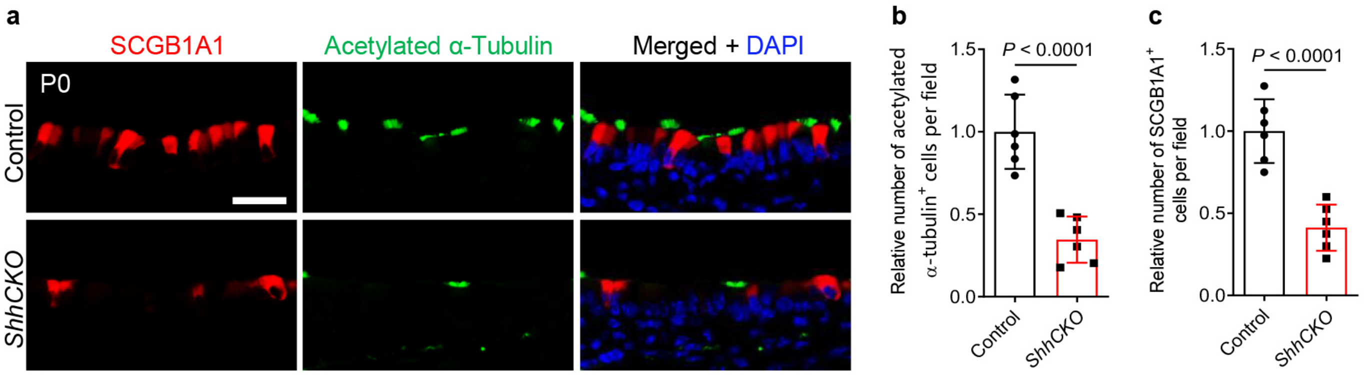

Fig. S8. *Nkx2.1^{Cre};Shh$^{flox/flox}$* mice exhibit defects in tracheal epithelial cell differentiation at P0.

(**a**) Immunostaining for SCGB1A1 (red), acetylated alpha-tubulin (green) and DAPI staining (blue) of longitudinal sections of P0 control (n=6) and *ShhCKO* (n=6) tracheae. (**b**) Quantification of the relative number of SCGB1A1^{+} cells in the tracheal epithelium in P0 control (n=6) and *ShhCKO* (n=6) mice. (**c**) Quantification of the relative number of acetylated alpha-tubulin^{+} cells in the tracheal epithelium in P0 control (n=6) and *ShhCKO* (n=6) mice. Scale bars: 50 µm. Unpaired Student's *t*-test, mean ± s.d.

Development • Supplementary information

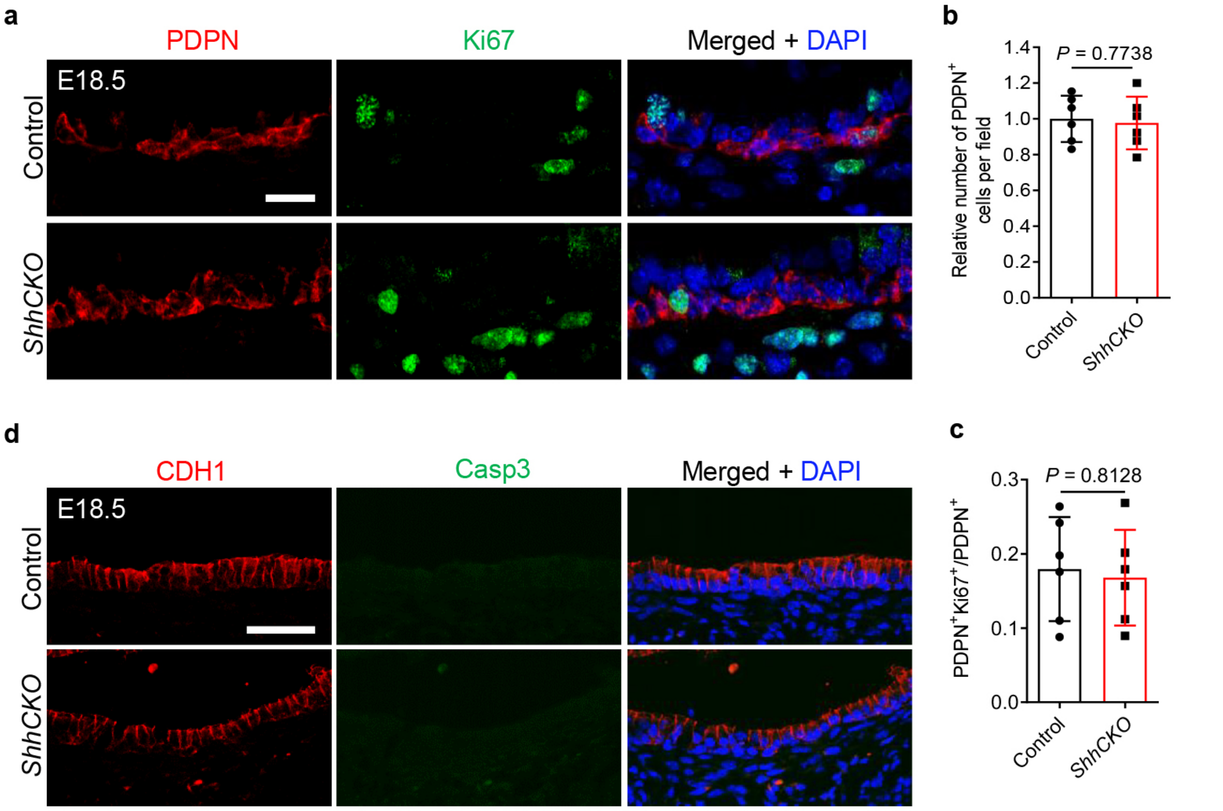

Fig. S9. *Nkx2.1^{Cre};Shh$^{flox/flox}$* mice exhibit WT-like proliferation rate of PDPN^{+} basal cells and apoptosis of CDH1^{+} cells in the trachea.

(**a**) Immunostaining for PDPN (red), Ki67 (green), and DAPI staining (blue) of longitudinal sections of E18.5 control (n=6) and *ShhCKO* (n=6) tracheae. (**b**) Quantification of the relative number of PDPN^{+} cells in the tracheal epithelium in E18.5 control (n=6) and *ShhCKO* (n=6) mice. (**c**) Ratio of PDPN^{+} cells that are Ki67^{+}. (**d**) Immunostaining for CDH1 (red), cleaved caspase-3 (Casp3, green) and DAPI staining (blue) of longitudinal sections of E18.5 control (n=6) and *ShhCKO* (n=6) tracheae. Scale bars: 50 μm (d), 20 μm (a). Unpaired Student's *t*-test, mean ± s.d.

Development • Supplementary information

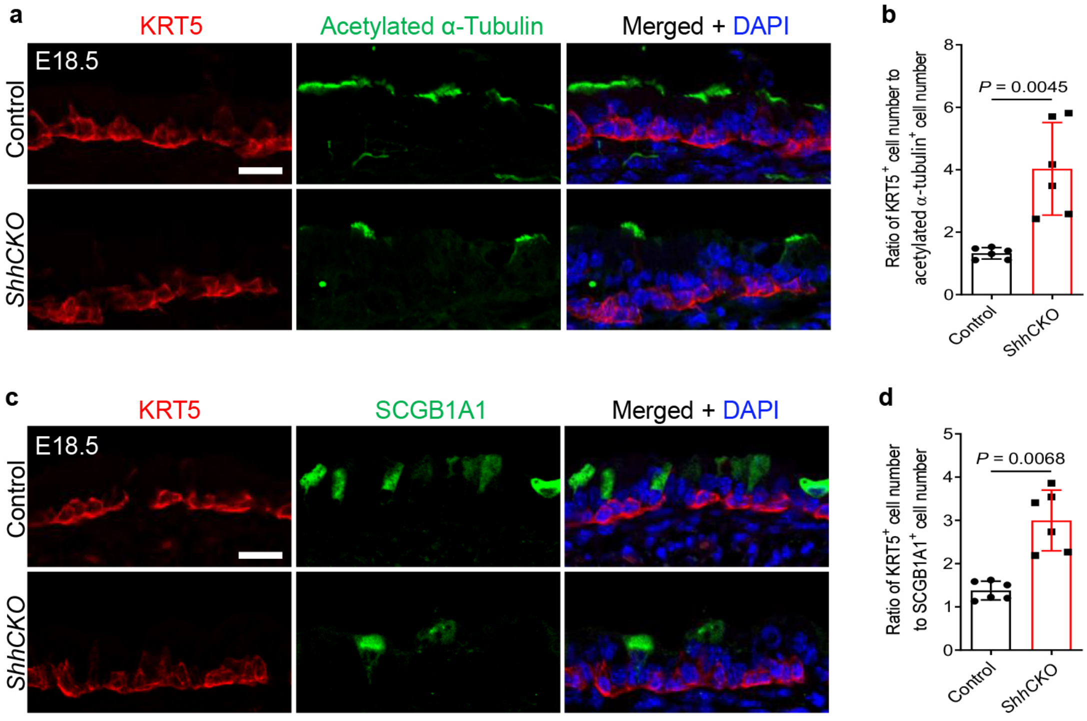

Fig. S10. ***Nkx2.1^{Cre};Shh$^{flox/flox}$*** **tracheae exhibit increased ratio of basal cells to ciliated and secretory cells.**

(**a**) Immunostaining for KRT5 (red) and acetylated alpha-tubulin (green) and DAPI staining (blue) of longitudinal sections of E18.5 control (n=6) and *ShhCKO* (n=6) tracheae. (**b**) Ratio of KRT5$^+$ cells to acetylated alpha-tubulin$^+$ cells. (**c**) Immunostaining for KRT5 (red) and SCGB1A1 (green) and DAPI staining (blue) of longitudinal sections of E18.5 control (n=6) and *ShhCKO* (n=6) tracheae. (**d**) Ratio of KRT5$^+$ cells to SCGB1A1$^+$ cells. Scale bars: 20 µm. Unpaired Student's *t*-test, mean ± s.d.

Development • Supplementary information

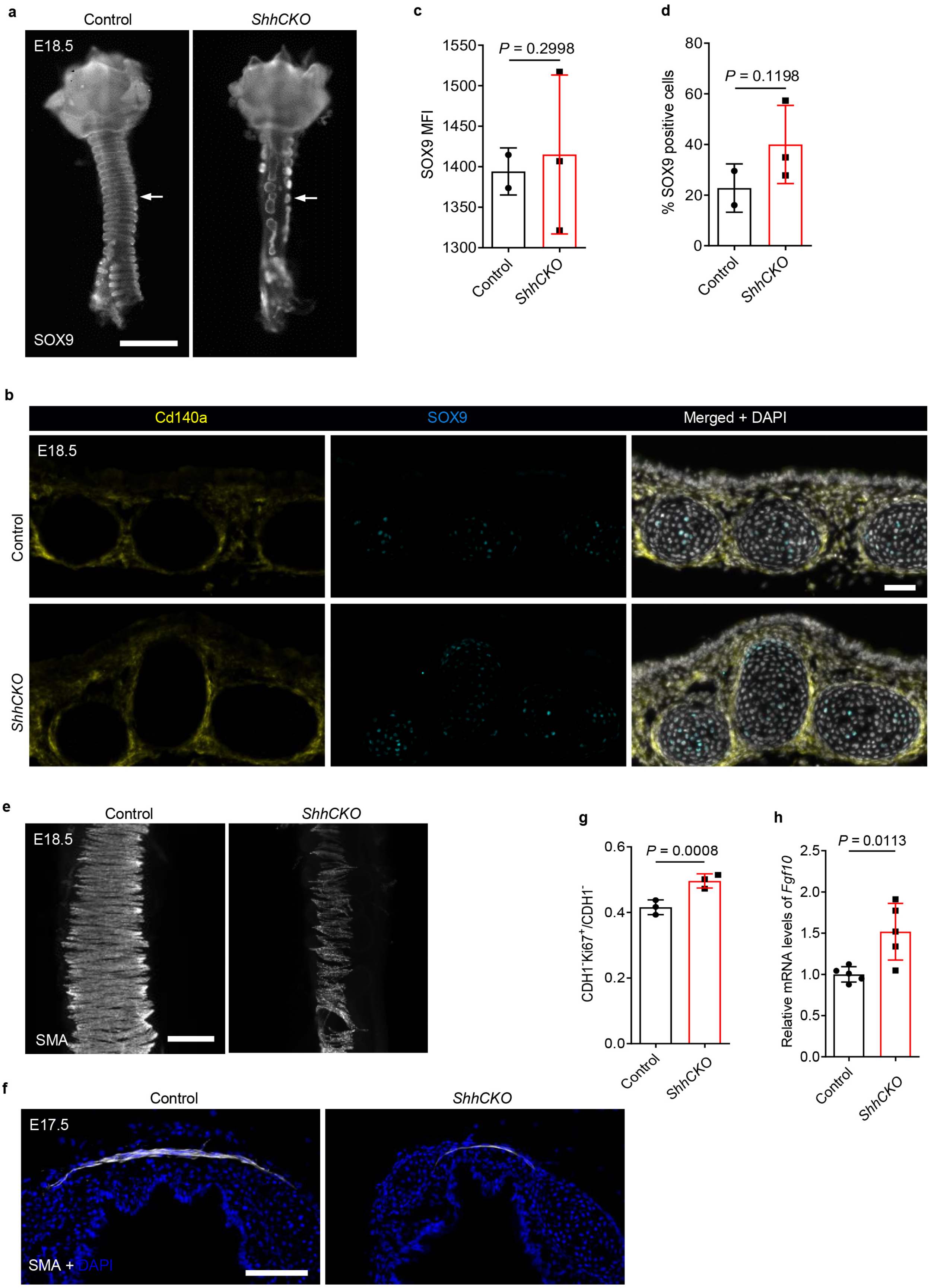

Development • Supplementary information

Fig. S11. *Nkx2.1^{Cre};Shh$^{flox/flox}$* tracheae exhibit defects in SOX9^{+} mesenchymal cell condensation and smooth muscle formation.
(**a**) Immunostaining for SOX9 (white) in ventral views of E18.5 control (n = 5) and *ShhCKO* (n = 5) tracheae. Arrows point to tracheal mesenchymal condensations. (**b**) Immunostaining for Cd140a (yellow) and SOX9 (turquoise) of longitudinal sections of E18.5 control and *ShhCKO* tracheae. (**c**) Quantification of mean fluorescent intensity (MFI) of SOX9 positive nuclei of E18.5 control (n=145) and *ShhCKO* (n=522) tracheae. (**d**) Quantification of the percentage of SOX9 positive nuclei in control (n=645) and *ShhCKO* (n=1302) tracheae. (**e**) Dorsal views of whole-mount tracheae stained for α-SMA from E17.5 control and *ShhCKO* mice. (**f**) Immunostaining for α-SMA (white) and DAPI staining (blue) of transverse sections of E18.5 control and *ShhCKO* tracheae. (**g**) Ratio of CDH1^{-}Ki67^{+} cells to CDH1^{-} cells in E18.5 control and *ShhCKO* tracheae. (**h**) RT–qPCR analysis of *Fgf10* mRNA levels in E18.5 control and *ShhCKO* tracheae. Scale bars: 1000 µm (**a**), 300 µm (**e**), 100 µm (**f**), 50 µm (**b**). Unpaired Student's *t*-test, mean ± s.d.

Development • Supplementary information

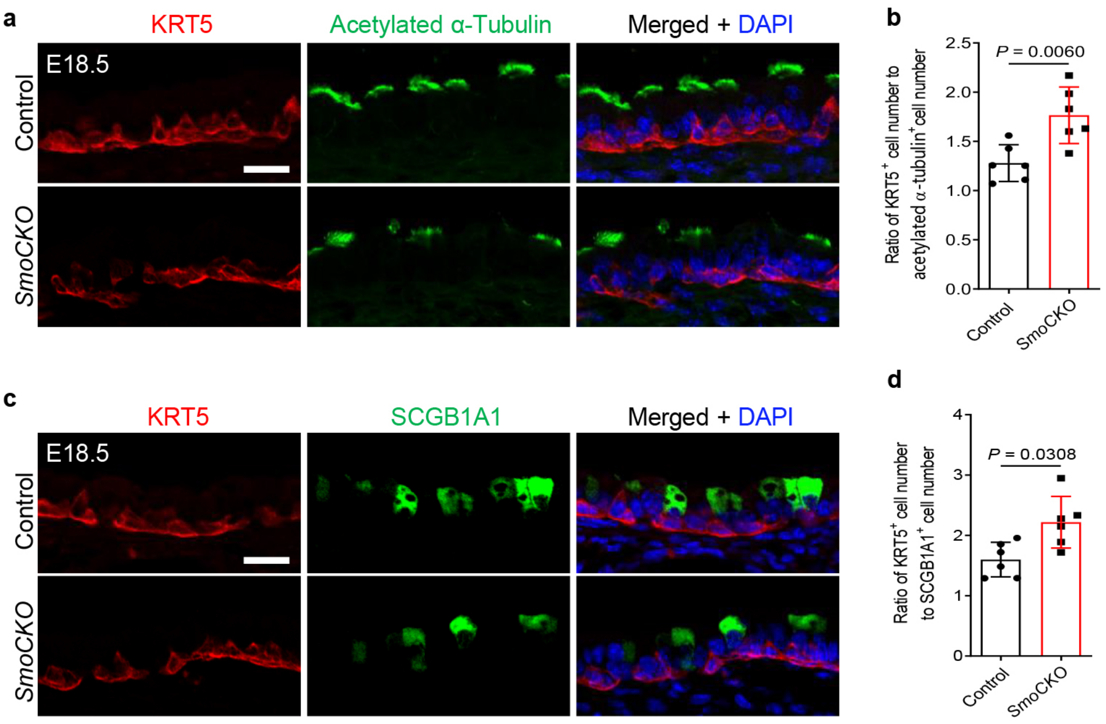

Fig. S12. ***Nkx2.1^{Cre};Smo$^{flox/flox}$*** **tracheae exhibit increased ratio of basal cells to ciliated and secretory cells.**

(**a**) Immunostaining for KRT5 (red) and acetylated alpha-tubulin (green) and DAPI staining (blue) of longitudinal sections of E18.5 control (n=6) and *SmoCKO* (n=6) tracheae. (**b**) Ratio of KRT5^{+} cells to acetylated alpha-tubulin^{+} cells. (**c**) Immunostaining for KRT5 (red) and SCGB1A1 (green) and DAPI staining (blue) of longitudinal sections of E18.5 control (n=6) and *SmoCKO* (n=6) tracheae. (**d**) Ratio of KRT5^{+} cells to SCGB1A1^{+} cells. Scale bars: 20 µm. Unpaired Student's *t*-test, mean ± s.d.

Development • Supplementary information

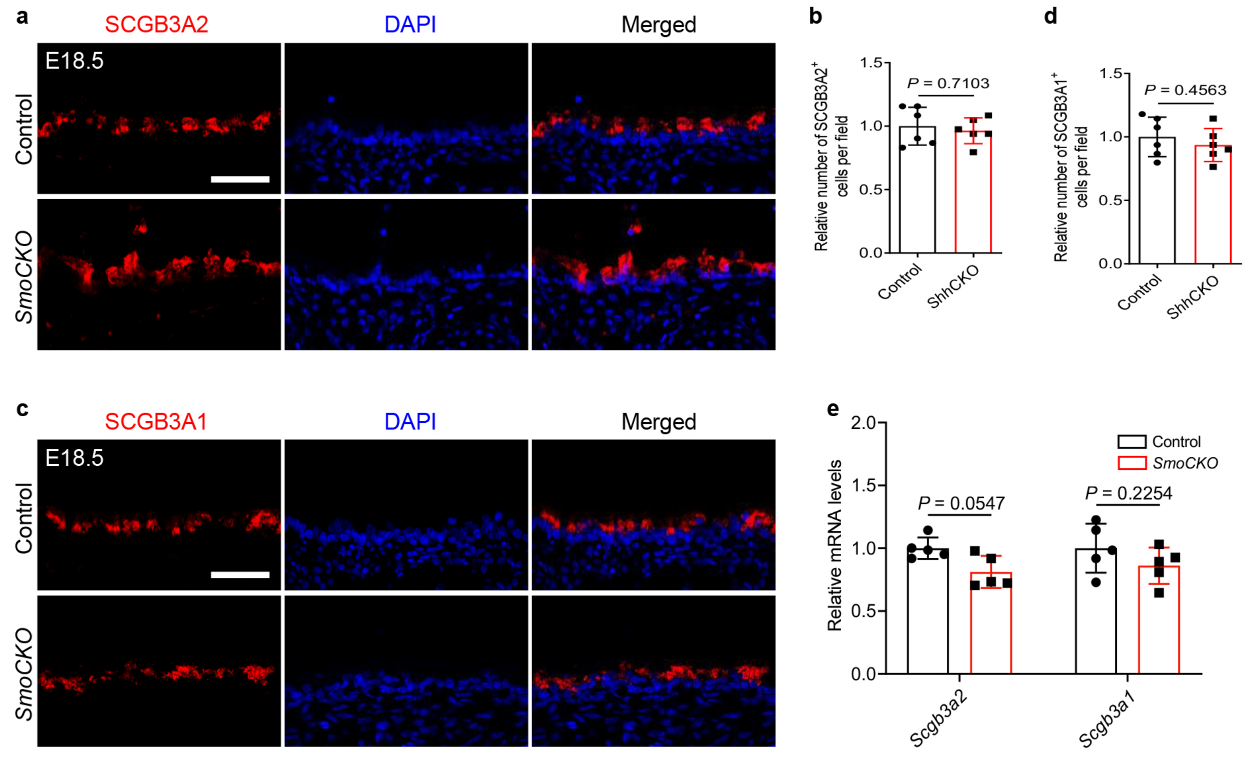

Fig. S13. Epithelial *Smo* deletion in *Nkx2.1*^{Cre}*;Smo*$^{flox/flox}$ mice does not affect the differentiation of SCGB3A2^{+} cells and SCGB3A1^{+} cells.

(**a**) Immunostaining for SCG32A2 (red) and DAPI staining (blue) of longitudinal sections of E18.5 control (n=6) and *SmoCKO* (n=6) tracheae. (**b**) Quantification of the relative number of SCGB3A2^{+} cells in the tracheal epithelium in control (n=6) and *SmoCKO* (n=6) mice. (**c**) Immunostaining for SCGB3A1 (red) and DAPI staining (blue) of longitudinal sections of E18.5 control (n=6) and *SmoCKO* (n=6) tracheae. (**d**) Quantification of the relative number of SCGB3A1^{+} cells in the tracheal epithelium in control (n=6) and *SmoCKO* (n=6) mice. (**e**) RT-qPCR analysis of *Scgb3a2* and *Scgb3a1* mRNA levels in E18.5 control (n=6) and *SmoCKO* (*n*=6) tracheae. Scale bars: 50 µm. Unpaired Student's *t*-test, mean ± s.d.

Development • Supplementary information

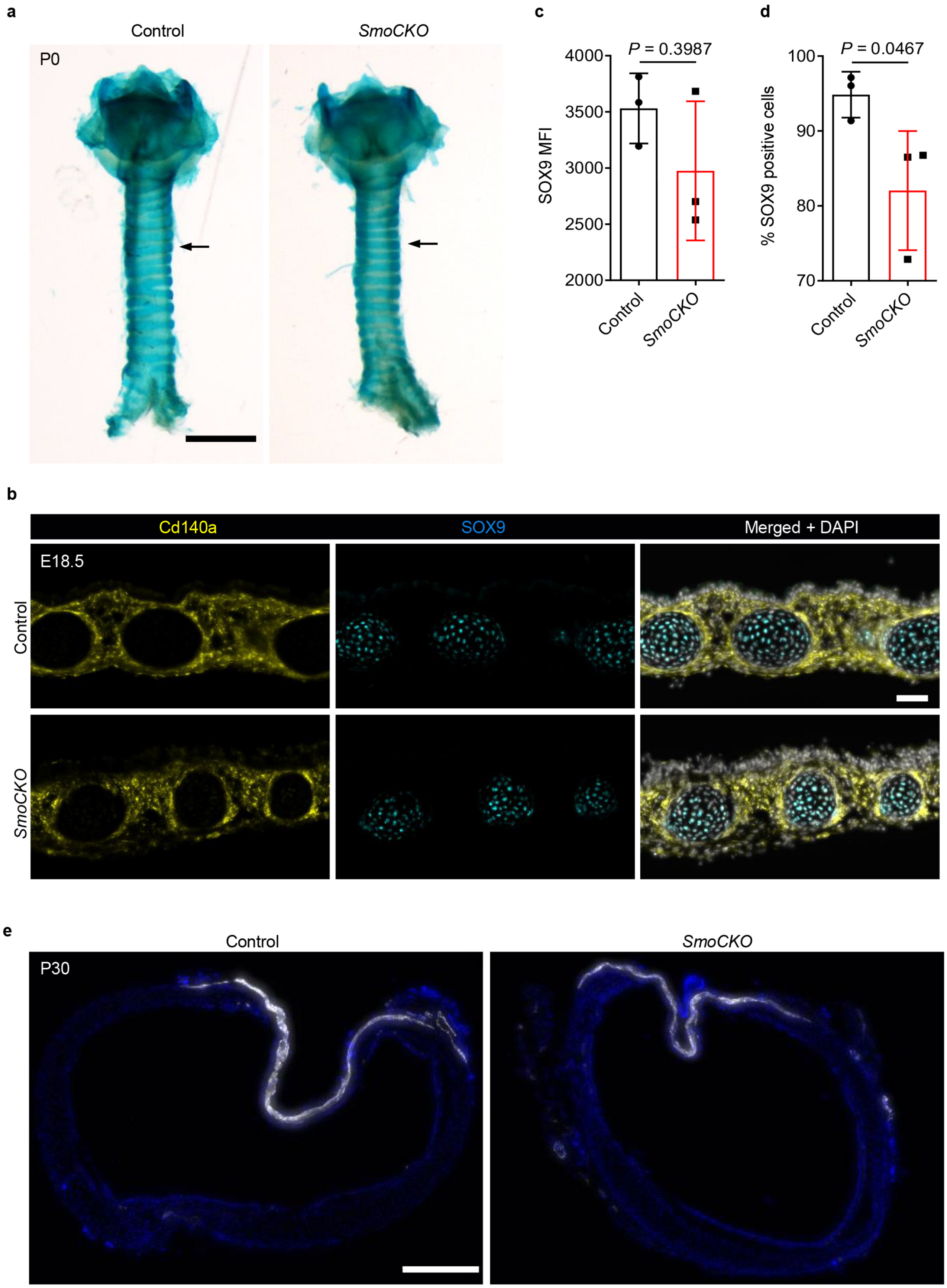

Development • Supplementary information

Fig. S14. ***Nkx2.1^{Cre};Smo$^{flox/flox}$*** **tracheae exhibit normal formation of cartilage rings and differentiation of chondroblasts and smooth muscle cells.**

(**a)** Representative images of ventral views of whole-mount tracheae stained with alcian blue from P0 control (*n* = 5) and *SmoCKO* mutants (*n* = 5). Arrows point to tracheal cartilage rings. (**b**) Immunostaining for Cd140a (yellow) and SOX9 (turquoise) of longitudinal sections of E18.5 control and *SmoCKO* tracheae. (**c**) Quantification of mean fluorescent intensity (MFI) of SOX9 positive nuclei of E18.5 control (n=907) and *SmoCKO* (n=824) tracheae. (**d**) Quantification of the percentage of SOX9 positive nuclei in control (n=955) and *SmoCKO* (n=1008) tracheae. (**e**) Immunostaining for α-SMA (white) and DAPI staining (blue) of transverse sections of P30 control and *SmoCKO* tracheae. Scale bars: 1000 µm (**a**), 200 µm (**e**), 50 µm (**b**). Unpaired Student's *t*-test, mean ± s.d.

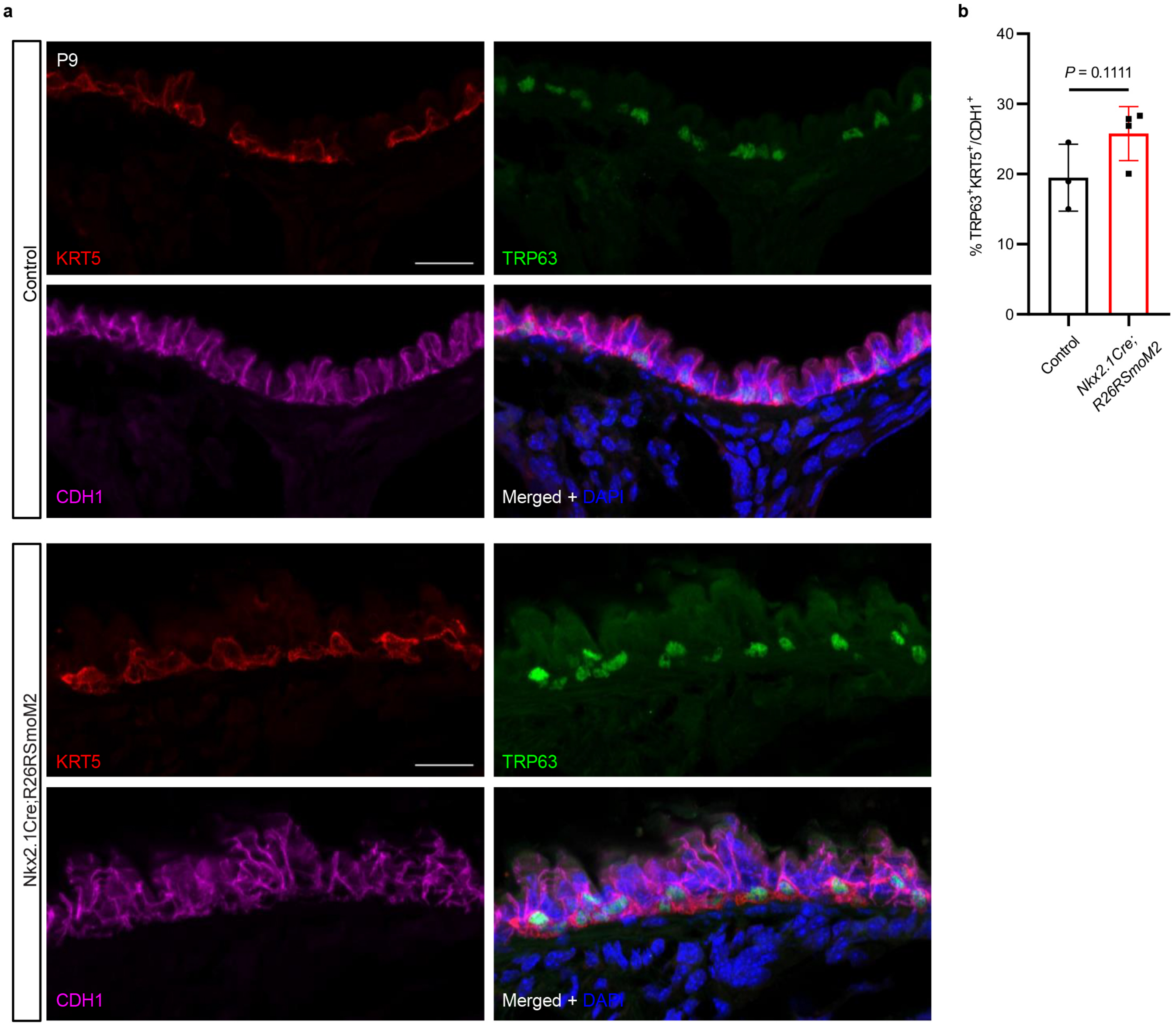

Fig. S15. Basal cells do not show significant difference after overactivation of *Smo*. (**a**) Immunostaining for KRT5 (red), TRP63 (green), CDH1 (magenta) and DAPI staining (blue) of longitudinal sections of P9 control (n=3) and *Nkx2.1*^{Cre};*R26*^{SmoM2}(n=4) mice. (**b**) Quantification of the percentage of TRP63^{+} and KRT5^{+} double positive cells in control (k=1649, n=3) and *Nkx2.1*^{Cre};*R26*^{SmoM2} (k=1916, n=4) mice. Scale bars: 20 µm. Unpaired Student's *t*-test, mean ± s.d.

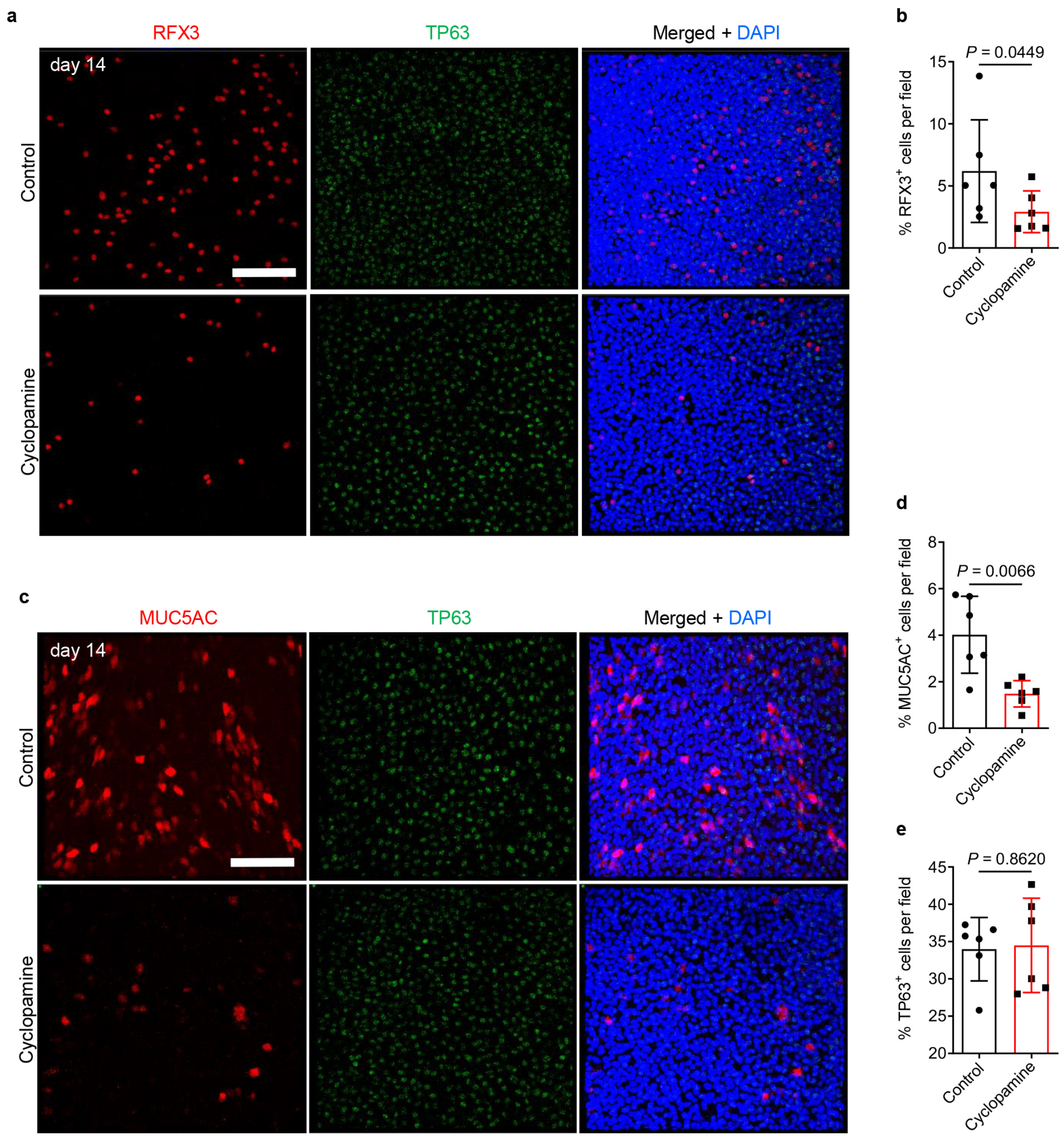

Fig. S16. Percentage of goblet cells and multiciliated cells but not basal cells is altered after Hh signaling inhibition during HBE cell differentiation.

(**a**) Immunostaining for RFX3 (red) and TP63 (green) and DAPI staining (blue) in HBE cells at the ALI after 9 days of ethanol (n=6) or 10 µM cyclopamine (n=6) treatment. (**b**) Percentage of RFX3^{+} cells. (**c**) Immunostaining for MUC5AC (red) and TP63 (green) and DAPI staining (blue) in HBE cells at the ALI after 9 days of ethanol (n=6) or 10 µM cyclopamine (n=6) treatment. (**d**) Percentage of MUC5AC^{+} cells. (**e**) Percentage of TP63^{+} cells. Scale bars: 100 µm. Unpaired Student's t-test, mean ± s.d.

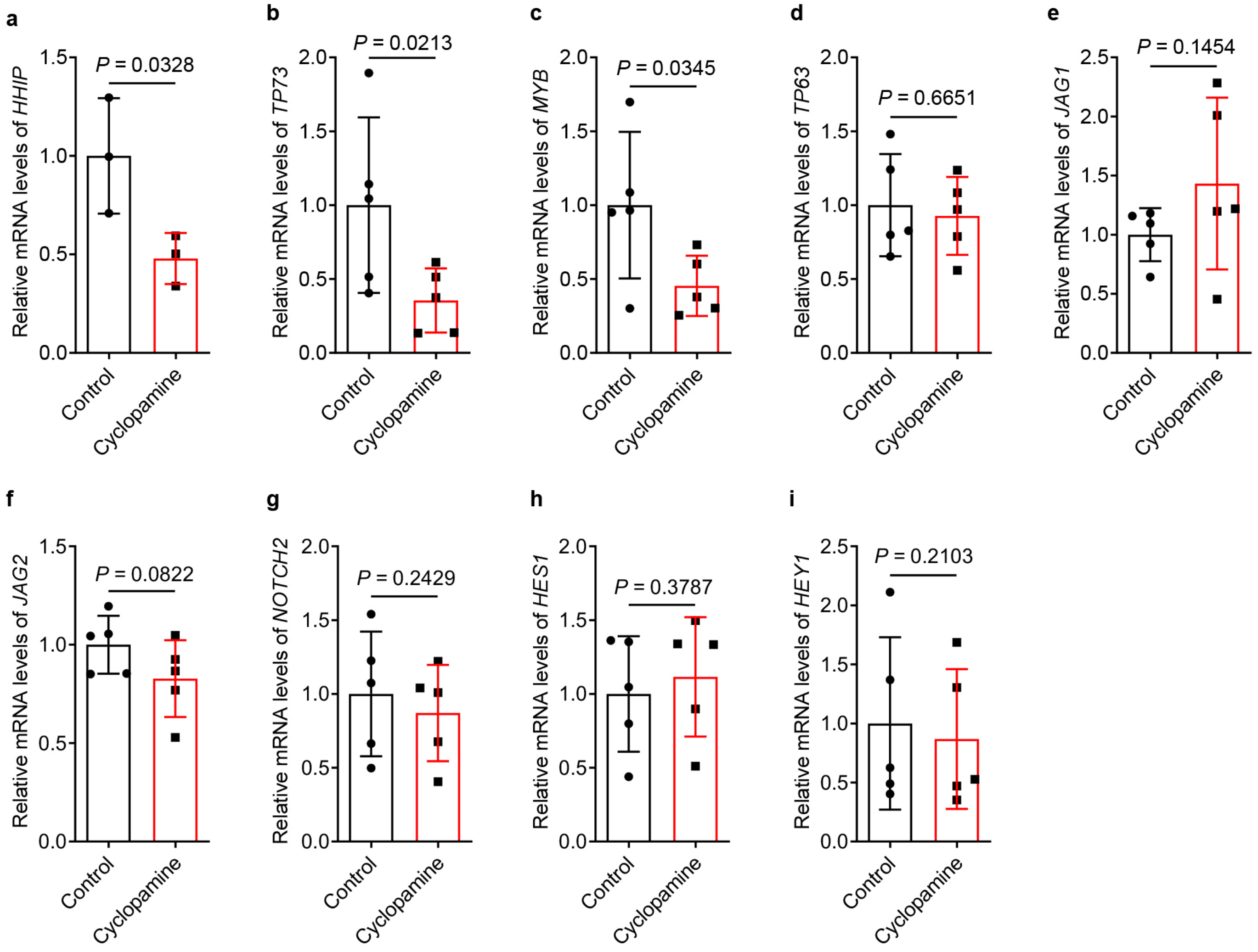

Fig. S17. Hh signaling inhibition leads to decreased *TP73* and *MYB* mRNA levels. **(a)** RT–qPCR analysis of *HHIP* mRNA levels in HBE cells at the ALI after 9 days of ethanol (n = 3) or 10 µM cyclopamine (n = 3) treatment. **(b-i)** RT–qPCR analysis of *TP73*, *MYB*, *TP63*, *JAG1*, *JAG2*, *NOTCH2*, *HES1*, and *HEY1* mRNA levels in HBE cells at the ALI after 9 days of ethanol (n = 5) or 10 µM cyclopamine (n = 5) treatment. Unpaired Student's *t*-test, mean ± s.d.

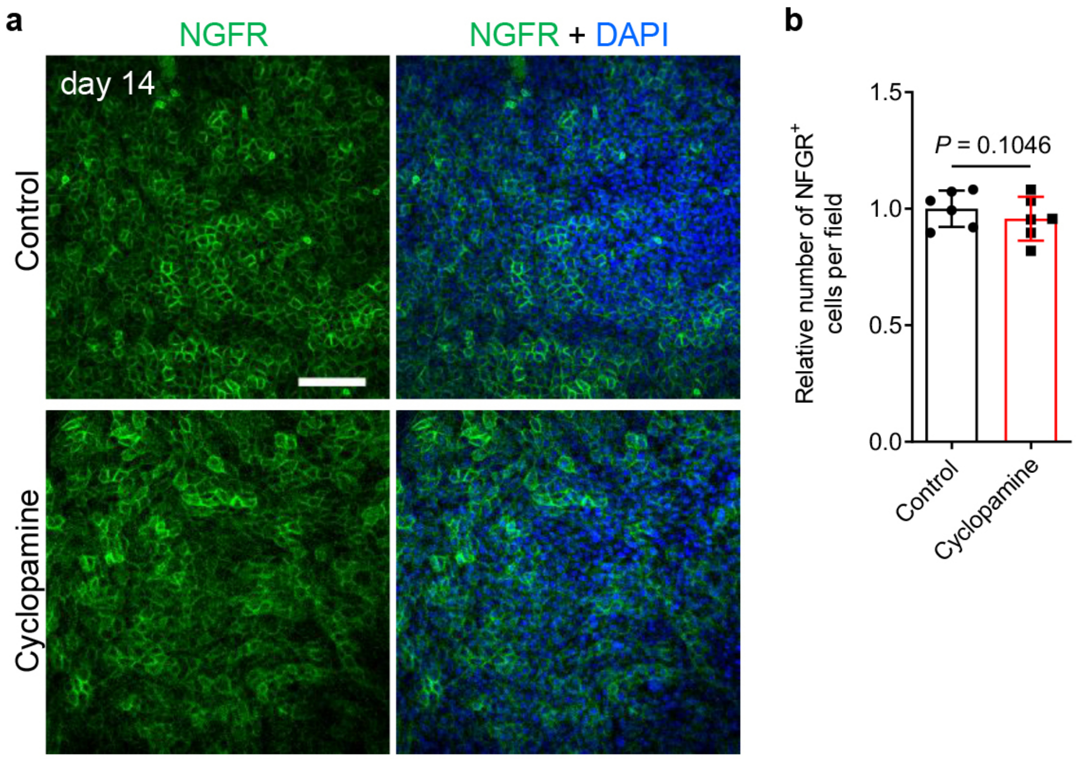

Fig. S18. Hh signaling inhibition did not affect the number of NGFR^{+} basal cells in ALI cultures.

(**a**) Immunostaining for NGFR (green) and DAPI staining (blue) in HBE cells at the ALI after 9 days of ethanol (n = 6) or 10 µM cyclopamine (n = 6) treatment. (**b**) Quantification of the relative number of NGFR^{+} cells in HBE cells at the ALI after 9 days of ethanol (n = 6) or 10 µM cyclopamine (n = 6) treatment. Scale bars: 100 µm. Unpaired Student's *t*-test, mean ± s.d.

Development • Supplementary information

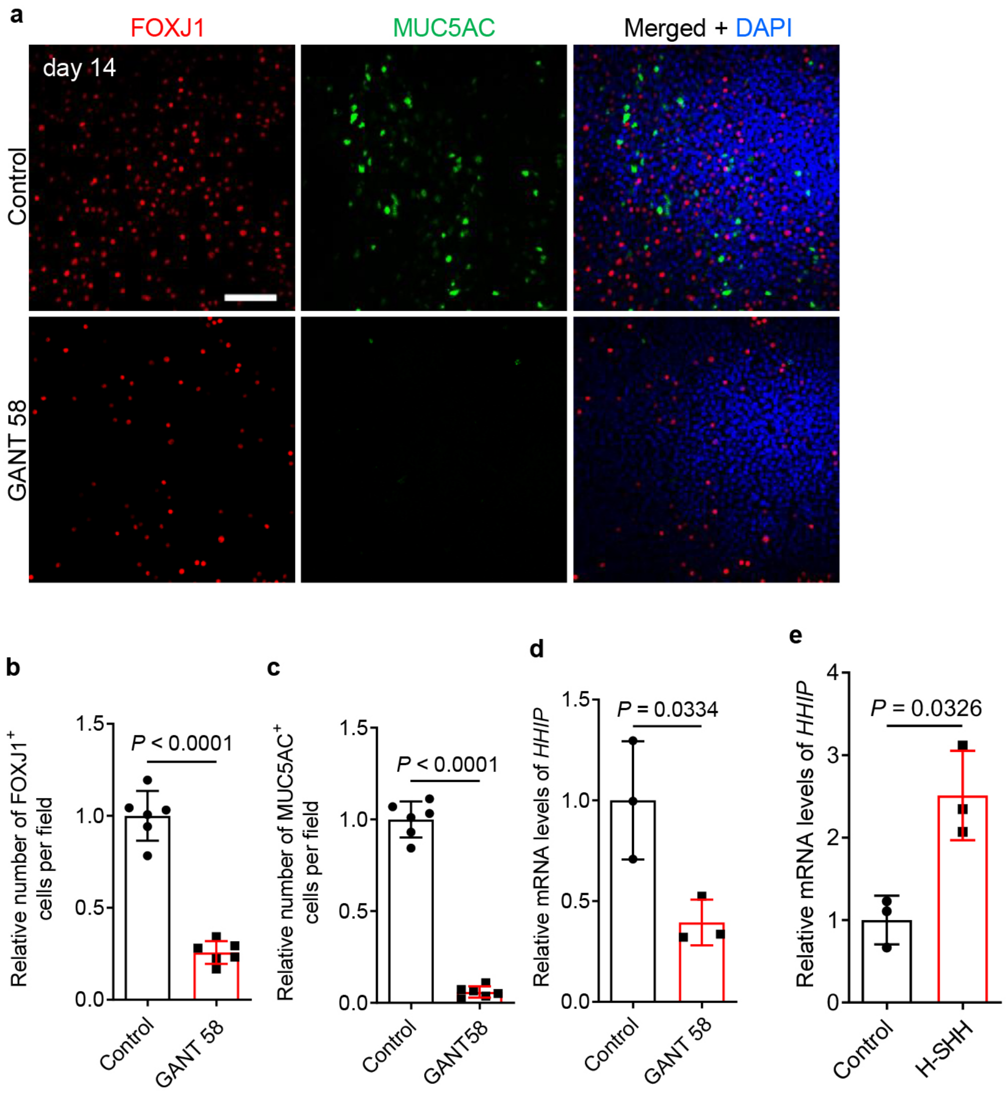

Fig. S19. GANT 58 treatment inhibited differentiation of HBE cells.

(**a**) Immunostaining for FOXJ1 (red), MUC5AC (green) and DAPI staining (blue) in HBE cells at the ALI after 9 days of ddH$_2$O (n = 6) or 10 μM GANT 58 (n = 6) treatment. (**b**) Quantification of the relative number of FOXJ1$^+$ cells in HBE cells at the ALI after 9 days of ddH$_2$O (n = 6) or 10 μM GANT 58 (n = 6) treatment. (**c**) Quantification of the relative number of MUC5AC$^+$ cells in HBE cells at the ALI after 9 days of ddH$_2$O (n = 6) or 10 μM GANT 58 (n = 6) treatment. (**d**) RT–qPCR analysis of *HHIP* mRNA levels in HBE cells at the ALI after 9 days of ddH$_2$O (n = 3) or 10 μM GANT 58 (n = 3) treatment. (**e**) RT–qPCR analysis of *HHIP* mRNA levels in HBE cells at the ALI after 9 days of ddH$_2$O (n = 3) or 100 ng/ml H-SHH (n = 3) treatment. Unpaired Student's *t*-test, mean ± s.d. Scale bars: 100 μm. Unpaired Student's *t*-test, mean ± s.d.

Development • Supplementary information

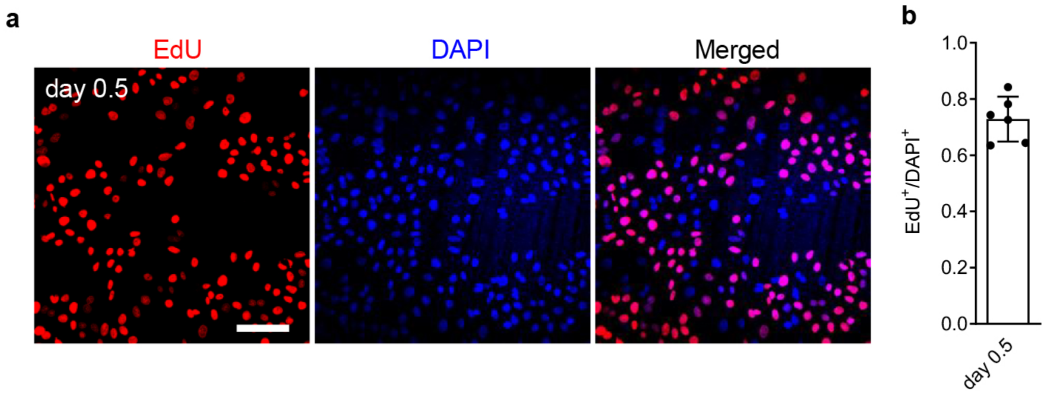

Fig. S20. EdU label most of HBE cells at day 0.5.

(**a**) EdU fluorescence (red) and DAPI staining (blue) in HBE cells at the ALI at day 0.5 (n=6). (**b**) Ratio of DAPI$^+$ cells that are EdU$^+$. Scale bars: 100 µm.

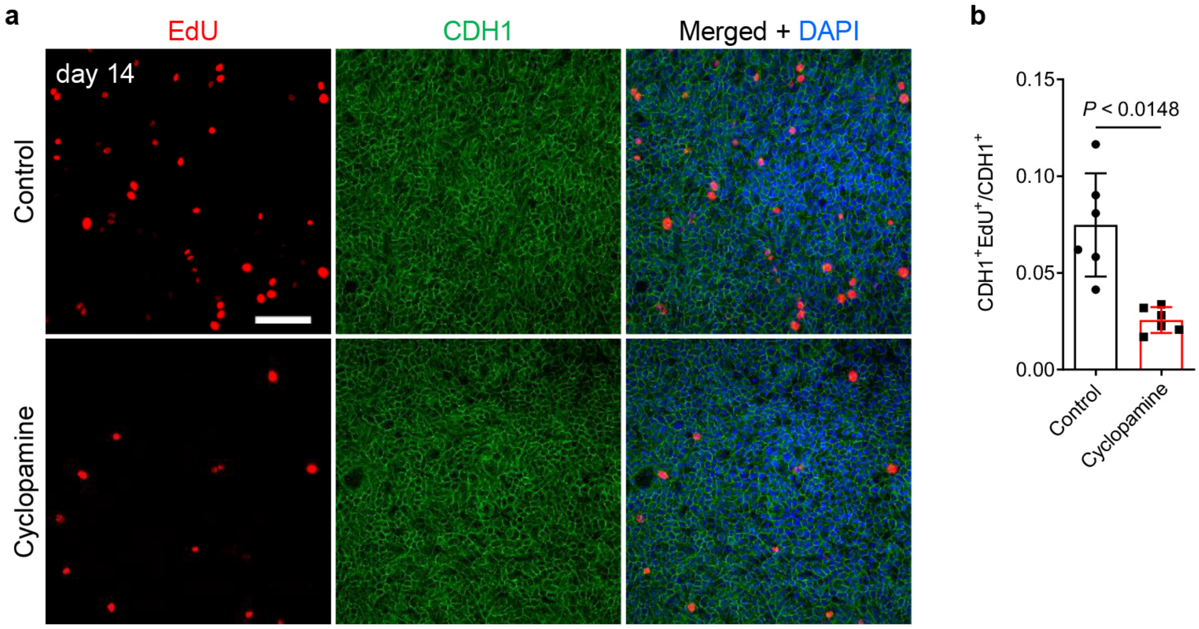

Fig. S21. Hh signaling inhibition compromised proliferation of CDH1$^+$ epithelial cells during HBE cell differentiation.

(**a**) EdU fluorescence (red), immunostaining for CDH1 (green) and DAPI staining (blue) in HBE cells at the ALI after 9 days of ethanol (n = 6) or 10 µM cyclopamine (n = 6) treatment. (**b**) Percentage of CDH1$^+$ cells that are EdU$^+$. Scale bars: 100 µm.

Development • Supplementary information

Table S1. Probe sequences for SCRINSHOT.

Click here to download Table S1

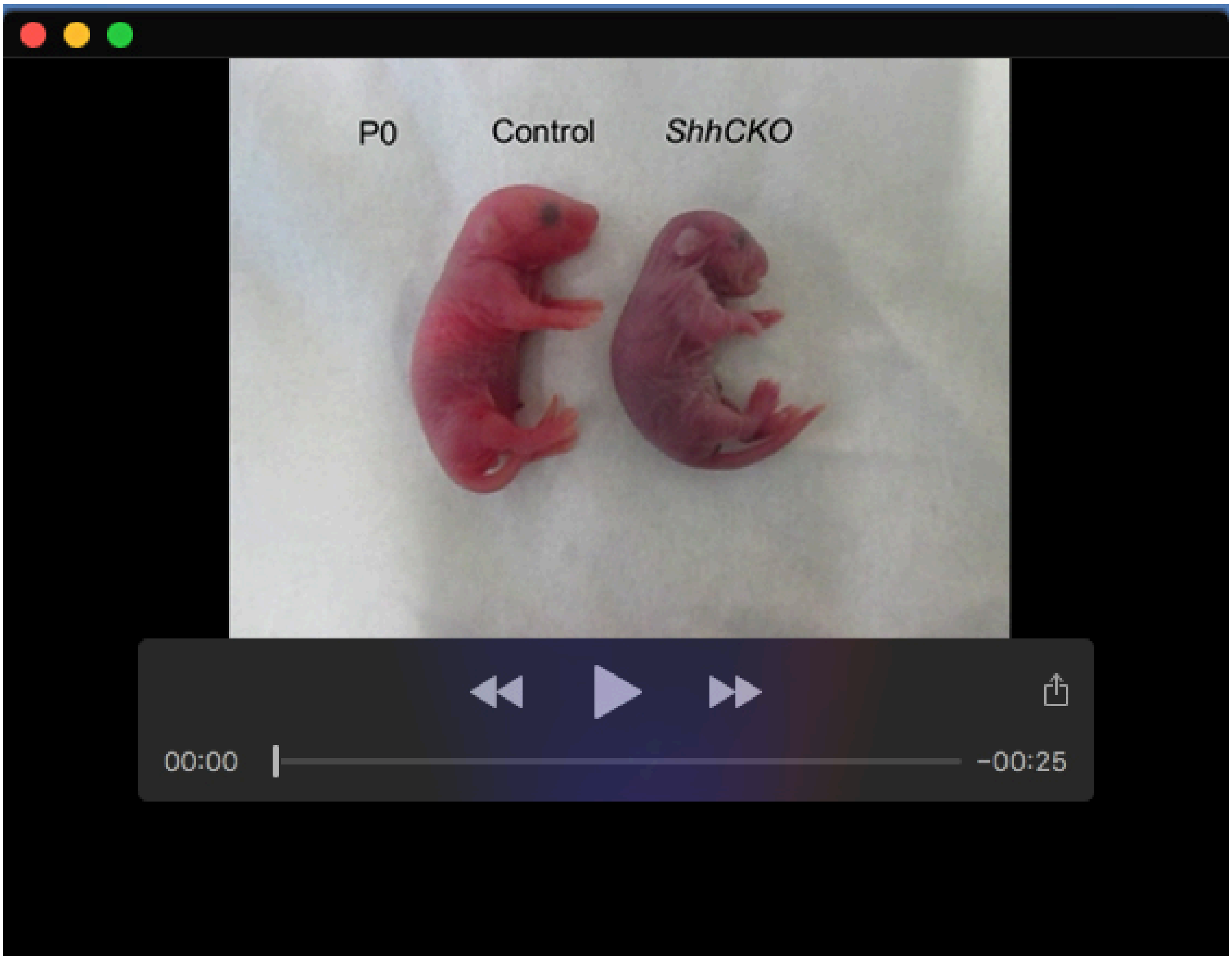

Movie 1. *ShhCKO* mice (n=6) exhibit neonatal respiratory distress.

Development • Supplementary information
